# Two Polarities of Attention in Social Contexts: From Attending-to-Others to Attending-to-Self

**DOI:** 10.3389/fpsyg.2016.00063

**Published:** 2016-02-01

**Authors:** Shenbing Kuang

**Affiliations:** State Key Laboratory of Brain and Cognitive Science, Institute of Psychology, Chinese Academy of SciencesBeijing, China

**Keywords:** social attention, attending-to-others, attending-to-self, neural mechanism, autism spectrum disorders, Williams syndrome

## Abstract

Social attention is one special form of attention that involves the allocation of limited processing resources in a social context. Previous studies on social attention often regard how attention is directed toward socially relevant stimuli such as faces and gaze directions of other individuals. In contrast to attending-to-others, a different line of researches has shown that self-related information such as own face and name automatically captures attention and is preferentially processed comparing to other-related information. These contrasting behavioral effects between attending-to-others and attending-to-self prompt me to consider a synthetic viewpoint for understanding social attention. I propose that social attention operates at two polarizing states: In one extreme, individual tends to attend to the self and prioritize self-related information over others', and, in the other extreme, attention is allocated to other individuals to infer their intentions and desires. Attending-to-self and attending-to-others mark the two ends of an otherwise continuum spectrum of social attention. For a given behavioral context, the mechanisms underlying these two polarities will interact and compete with each other in order to determine a saliency map of social attention that guides our behaviors. An imbalanced competition between these two behavioral and cognitive processes will cause cognitive disorders and neurological symptoms such as autism spectrum disorders and Williams syndrome. I have reviewed both behavioral and neural evidence that support the notion of polarized social attention, and have suggested several testable predictions to corroborate this integrative theory for understanding social attention.

## Behavioral evidence of polarized social attention

Humans are exceptionally social species and we constantly pay close attention to the intentions and desires of other individuals when we interact in a social setting (Klein et al., 2009). Such behavioral tendency of attending-to-others allows us to infer the mental states of others and therefore respond in a context-appropriate manner. Of the socially relevant stimuli, faces and gazes are the two most important elements. For instance, we can readily recognize people's emotions from their facial expressions (Atkinson and Adolphs, 2011), and we often reflexively follow other individuals' gaze directions to attend to the same locations or events (Driver et al., 1999; Ricciardelli et al., 2002; Friesen and Kingstone, 2003; Tipples, 2005; for a review, see Frischen et al., 2007). Other social cues, like head orientation (Langton and Bruce, 1999) and walking direction of living organisms (Blake and Shiffrar, 2007), have also been shown to attract our attention automatically. Attending-to-others has been the focus of a large number of studies in the past few years (Itier and Batty, 2009; Klein et al., 2009; Skarratt et al., 2012) and it is considered to be a fundamental cognitive function for any social species. Malfunctions of attending-to-others will cause significant deficits in social interaction and social communication (Guillon et al., 2014). One such example is patients with autism spectrum disorders (ASD) who tend to spend less time attending to socially salient features, such as faces and eyes of other individuals (Sigman et al., 2006), and a failure to make use of these information for mind-reading (Spezio et al., 2007). Opposite to reduced attending-to-others in ASD patients, patients with Williams syndrome (WS) often exhibit excessive attending-to-others: they are eagerly engaged in social interaction and show unusually high empathy for others (Järvinen et al., 2013).

In parallel to the above attending-to-others and other-centered perspective, attending to the bodily self is also of survival importance in our daily life. Self-related stimuli such as one's own name and face are arguably the most familiar and critical information for us and, consequently, they have processing advantages and can also capture our attention without awareness (Humphreys and Sui, 2015). Increasing evidence has indicated that perception and attention are biased toward self-related stimuli. For example, face orientation discrimination is faster and more accurate when judging your own face comparing to others' faces (Ma and Han, 2010; Keyes and Dlugokencka, 2014); participants are faster and more accurate in a visual search task when the search targets are their own names relative to the names of other individuals (Harris et al., 2004; Yang et al., 2013). Similar self-related behavioral advantages have also been reported for the recognition of both static and moving body parts (e.g., foot, hand; Frassinetti et al., 2008, 2009).

Taken together, the contrasting behavioral effects between attending-to-others and attending-to-self seem to dispute the simplified view that resources of attention in social contexts are oriented merely toward others (Klein et al., 2009) or are anchored exclusively to self-related representations (Humphreys and Sui, 2015). Instead, attention in social contexts is a dynamic behavioral and cognitive process that could be flexibly employed to enhance any behaviorally salient stimuli ranging from the self to the others, depending on specific social contexts. In other words, social attention is not a fixed property of the cognitive system. For each given behavioral context, the self-centered aspect and other-centered aspect will interact and compete with each other for the sake of deciding a saliency map of attention in social contexts that dictates how attention resources will be distributed among the two aspects. As such, similar to the general attention systems (Corbetta and Shulman, 2002; Bisley and Goldberg, 2010), social attention can also be flexibly oriented according to a context-contingent saliency map. Ultimately, the saliency map of social attention will be integrated with the general attention control systems to guide our perception and action (Humphreys and Sui, 2015).

## Neural basis of polarized social attention

Attending-to-others recruits a widely distributed neural network that includes brain areas implicated in face and gaze perception, i.e., fusiform gyrus (Haxby et al., 2000), posterior portion of superior temporal sulcus (pSTS; Allison et al., 2000; Nummenmaa and Calder, 2009), and brain areas specialized for emotion processing, e.g., the amygdale (Adolphs and Spezio, 2006), as well as parietal-frontal areas that are dedicated to theory of mind and social cognition, i.e., temporal-parietal junction (TPJ; Gallese and Goldman, 1998; Decety and Jackson, 2004), medial prefrontal cortex (mPFC; Amodio and Frith, 2006; Lieberman, 2007). Individuals with abnormal levels of attending-to-others, either insufficient (ASD) or excessive (WS), often exhibit functional and structural impairments within these neural structures (Pelphrey et al., 2004; Amaral et al., 2008; Järvinen et al., 2013).

On the other hand, self-reflection and attending-to-self have been tightly linked to area mPFC (Amodio and Frith, 2006; Lieberman, 2007). fMRI studies in healthy participants show that area mPFC is more activated when making judgments about self-related information relative to those of another person, irrespective of whether the information refers to veridical attributes such as personality traits and feelings (Jenkins and Mitchell, 2011), or arbitrary associations (e.g., associate a square with yourself, a triangle with strangers, and a circle with friends; Sui et al., 2013). In the latter study, beyond enhanced responses in mPFC, self-related stimuli also preferentially activate brain region pSTS—an area that has previously been associated with the functions of attending-to-others (Allison et al., 2000; Nummenmaa and Calder, 2009).

Notably, the brain regions responsible for attending-to-self and attending-to-others are partially overlapping, implying that the two aspects are not unconnected cognitive processes. Rather, it suggests that attending-to-self and attending-to-others should be viewed as two interdependent cognitive processes that are likely mediated via an integrative mechanism and theory. Intriguingly, while both mPFC and pSTS show enhanced fMRI responses to self-related stimuli (relative to other-related ones), the general activation patters in these two areas are opposite, i.e., the estimated beta values from general linear model analysis of fMRI signals are positive in one area but negative in the other (Sui et al., 2013). This means, area mPFC and area pSTS are responding oppositely (activation vs. inhibition) in this task, indicating that while both areas are involved in the processing of self- vs. others- related information, their specific roles might vary radically and contrast each other. One overarching explanation for the contrasting role is that area pSTS is primarily responsible for the functions of attending-to-others while area mPFC mainly supports the functions of attending-to-self. Neural interactions between these two areas will determine where exactly in the saliency map lies the current state of attention for a given social context. Remarkably, this interpretation has been recently supported by a neuropsychological study in patients with brain lesions to mPFC and pSTS, respectively. It shows that damage to mPFC leads to reduced attending-to-self comparing to healthy controls, while pSTS impairment results in increased attending-to-self (Sui et al., 2015).

## Implications and predictions

The concept that attending-to-self and attending-to-others mark two polarizing states of attention in social contexts unifies two complementary lines of researches in the field. It provides a synthetic and compelling framework for understanding social attention, not only in healthy humans but also in clinical populations with social attention deficits. Despite that a number of existing behavioral and neural studies have shown results compatible with this view, the previous studies are conducted in isolation, i.e., examining the properties of attending-to-self but not attending-to-others, and vice versa. Admittedly, it is not the obligation of previous studies to consider the two aspects in tandem, because there was no synthetic view suggesting such a linkage. But for future studies, it will be important to take an integrative perspective and implement task designs that will take into account both attending-to-self and attending-to-others at the same time. The integrative approach will likely unveil new important phenomena and findings that could not be discerned when considering these two aspects in isolation. In the following, I will provide several examples of experimental paradigms and specific predictions, which might be used to test and refine the ideas of polarized social attention.

First, the levels of attending-to-self, measured by advantages in the processing of self-related information (e.g., reduced reaction time), should be negatively correlated with the levels of attending-to-others (e.g., gaze following, time of eye-contact). For instance, individuals with greater self-related advantage will tend to show less frequent gaze following and spent less time maintaining eye-contact with other people. Second, manipulating social context should modulate the levels of attending-to-self and attending-to-others in a predictable way. For example, self-related advantages measured when participants are alone will be different from when they are surrounded by people but informed that others' behaviors are irrelevant to their tasks at hand. Third, behavioral interactions between attending-to-self and attending-to-others should be compared directly with their neural interactions in the respective brain structures mentioned above. Furthermore, the comparisons between behavioral and neural aspects need to be made not only in healthy human participants but also in patients with abnormal social attention functions. Ultimately, our goal is that the knowledge gained from these basic researches will be used for developing and improving strategies for treating clinical populations with impaired social functions.

## Author contributions

The author confirms being the sole contributor of this work and approved it for publication.

### Conflict of interest statement

The author declares that the research was conducted in the absence of any commercial or financial relationships that could be construed as a potential conflict of interest. The reviewer, Francois Quesque, and handling Editor declared their shared affiliation, and the handling Editor states that the process nevertheless met the standards of a fair and objective review.
